# Work difficulties in people with multiple sclerosis: The role of anxiety, depression and coping

**DOI:** 10.1177/20552173221116282

**Published:** 2022-09-04

**Authors:** EEA van Egmond, K van der Hiele, DAM van Gorp, PJ Jongen, JJL van der Klink, MF Reneman, EAC Beenakker, JJJ van Eijk, STFM Frequin, K de Gans, BM van Geel, OHH Gerlach, GJD Hengstman, JP Mostert, WIM Verhagen, HAM Middelkoop, LH Visser

**Affiliations:** Department of Neurology, 7898 Elisabeth-TweeSteden Hospital, Tilburg, the Netherlands; Institute of Psychology, Health, Medical and Neuropsychology Unit, 4496 Leiden University, Leiden, the Netherlands; National Multiple Sclerosis Foundation, Rotterdam, the Netherlands; Department of Care Ethics, University of Humanistic Studies, Utrecht, the Netherlands; Institute of Psychology, Health, Medical and Neuropsychology Unit, 4496 Leiden University, Leiden, the Netherlands; Department of Care Ethics, University of Humanistic Studies, Utrecht, the Netherlands; MS4 Research Institute, Nijmegen, the Netherlands; Department of Community & Occupational Medicine, University of Groningen, University Medical Centre Groningen, Groningen, the Netherlands; Tilburg School of Social and Behavioural Sciences, Tranzo Scientific Centre for Care and Welfare, Tilburg University, Tilburg, the Netherlands; Optentia, North West University of South Africa, Vanderbijlspark, South Africa; Department of Rehabilitation Medicine, Centre for Rehabilitation, University of Groningen, University Medical Centre Groningen, Haren, the Netherlands; Department of Neurology, 4480 Medical Centre Leeuwarden, Leeuwarden, the Netherlands; Department of Neurology, Jeroen Bosch Hospital, ‘s-Hertogenbosch, the Netherlands; Department of Neurology, St Antonius Hospital, Nieuwegein, the Netherlands; Department of Neurology, Groene Hart Hospital, the Netherlands; Department of Neurology, 1140 NoordWest Ziekenhuisgroep, the Netherlands; Department of Neurology, Zuyderland Medical Centre, Sittard-Geleen, the Netherlands; Department of Neurology, Maastricht University Medical Centre, Maastricht, the Netherlands; Department of Neurology, School for Mental Health and Neuroscience, Maastricht University Medical Centre, Maastricht, the Netherlands; Upendo MS Clinic, Boxtel, the Netherlands; Department of Neurology, Rijnstate Hospital, the Netherlands; Department of Neurology, Canisius-Wilhelmina Hospital, Nijmegen, the Netherlands; Institute of Psychology, Health, Medical and Neuropsychology Unit, 4496 Leiden University, Leiden, the Netherlands; Department of Neurology & Neuropsychology, 4496 Leiden University Medical Centre, Leiden, the Netherlands; Department of Neurology, 7898 Elisabeth-TweeSteden Hospital, Tilburg, the Netherlands; Department of Care Ethics, University of Humanistic Studies, Utrecht, the Netherlands

**Keywords:** multiple sclerosis, employment, work, depression, anxiety, coping behaviour

## Abstract

**Background:**

Symptoms of anxiety and depression affect the daily life of people with multiple sclerosis (MS). This study examined work difficulties and their relationship with anxiety, depression and coping style in people with MS.

**Methods:**

219 employed people with MS (median age  =  43 years, 79% female) completed questionnaires on anxiety, depression, coping style, demographics and work difficulties, and underwent a neurological examination. Two regression analyses were performed with work difficulties as the dependent variable and either anxiety or depression as continuous independent variables. Coping style, age, gender, educational level, MS-related disability and disease duration were added as additional predictors, as well as interaction terms between coping style and either symptoms of depression or anxiety.

**Results:**

A significant model was found (*F*_(10,205)_  =  13.14, *p* < 0.001, *R*^2^  =  0.39) in which anxiety, emotion- and avoidance-oriented coping and MS-related disability were positively related to work difficulties. The analysis of depression resulted in a significant model (*F*_(10,205)_  =  14.98, *p* < 0.001, *R*^2^  =  0.42) in which depression, emotion- and avoidance-oriented coping and MS-related disability were positively related to work difficulties. None of the interaction effects were significant.

**Conclusions:**

Work difficulties were positively related to anxiety, depression, emotion- and avoidance-oriented coping and MS-related disability in workers with MS.

## Introduction

Anxiety and depression are common mental health complaints in people with MS. Research has shown that depression and anxiety occur respectively in 31% and 22% of people with MS.^1^ This weighted prevalence is elevated compared to both the general population and people with other long-term medical conditions.^1,2^

Symptoms of depression and anxiety have debilitating implications for occupational functioning and have been associated with unemployment^3,4^ and the need to reduce work hours in people with MS.^5,6^ However, the literature evaluating the relationship between occupational functioning and symptoms of anxiety and depression remains equivocal, with other studies not being able to replicate this effect.^7–9^ Further examining correlates of occupational functioning is crucial, as decreased occupational functioning in MS is associated with high costs for the individual with MS,^10^ as well as high socio-economic costs.^11^ Additionally, being employed provides a professional identity and social interactions^12^ and is associated with life satisfaction.^4^

Recent studies have explored work difficulties in MS using the Multiple Sclerosis Work Difficulties Questionnaire (MSWDQ).^13,14^ Work difficulties are an important indicator of occupational functioning. Experiencing work difficulties is associated with reduced work hours after diagnosis, reduced expectations of future occupational functioning and a decrease in work status.^13,15^ Prior research has positively associated work difficulties with depressive symptoms,^13,15^ but the relationship between work difficulties (as measured with the MSWDQ) and anxiety has only been examined once.^16^ Van der Hiele et al.^16^ observed a positive relationship between anxiety and more work difficulties. In general, research on anxiety symptoms and its risk factors in MS is scarce,^17^ but separate studies do show associations between work outcomes and anxiety.^3,4^

A recent meta-analytic review^4^ identified the need for additional research inquiring into coping and employment in MS. Coping has been previously linked to employment status in people with MS. Avoidant coping styles, such as behavioural disengagement and denial, were associated with unemployment or shorter time to unemployment.^18–21^ Alternatively, employed people with MS tend to adopt more problem-focused coping strategies.^4,12^ Problem-focused coping is defined as ‘the use of active strategies to directly address the source of stress, challenge or problem faced.’^12^ In other literature, this coping style is also referred to as active coping or task-oriented coping.

Research in healthy workers (without MS) shows that active coping is associated with better work ability, while avoidant coping is associated with worse work ability.^22^ Additionally, they showed that coping styles may also moderate the relationship between mental health and occupational functioning.^22^ The latter study suggests that avoidant coping moderates the relationship between mental health and work ability: The positive association between mental health and work ability was stronger for people using more avoidant coping. The effect was small however. Additionally, a small moderation effect of active coping was found in the relationship between mental health and work ability, in that the usage of more active coping strengthened the association between mental health and work ability.^22^ To the best of our knowledge, this effect has not been examined in people with MS.

In this study, we investigated the association between symptoms of depression and anxiety and work difficulties. We hypothesised that more symptoms of depression and anxiety are associated with more work difficulties.^15^ Additionally, we expect a positive relationship between emotion- and avoidance-oriented coping and a negative relationship between task-related coping and work difficulties.^22^ Furthermore, we hypothesised that coping style moderates the relationship between either depression and work difficulties or anxiety and work difficulties.^22^ In line with previous research,^22^ we expect that the positive association between either anxiety or depression and work difficulties will be moderated by the usage of avoidance-oriented coping, in that the positive association will be stronger for people using more avoidant coping. Similarly, we expect that task-oriented coping and emotion-oriented coping will moderate the positive association between either depression or anxiety and work difficulties, in that the positive association will be stronger for people using more task-oriented coping and for people using more emotion-oriented coping.

## Methods

### Participants

We included 239 employed people with MS who participated in the MS@Work study.^23^ They completed questionnaires about demographic characteristics, psychological and occupational functioning, and took part in a neurological examination. Inclusion criteria, for the MS@Work study in general, were a diagnosis of relapsing-remitting MS in accordance with the Polman-McDonald criteria,^24^ and being employed or being within three years since the last employment. The minimum age for participation was 18 years. People with co-morbid psychiatric and/or other neurological disorders were not approached to participate (diagnosis according to the Diagnostic and Statistical Manual of Mental Disorders -fifth edition (DSM-5).^25^ Similarly, participants who dealt with substance abuse or neurological impairment that might interfere with cognitive testing, such as visual problems, were not approached to participate. All participants were fluent in the Dutch language. The study was approved by the Medical Ethical Committee Brabant (NL43098.008.12 1307). All participants signed an informed consent form before participating in the study, and the study was performed in agreement with the Declaration of Helsinki.

### Measures

#### Depression and anxiety symptoms

Symptoms of depression and anxiety were examined using the Hospital Anxiety and Depression Scale (HADS).^26^ The HADS is a 14-item scale for the screening of depression and anxiety. The scale consists of seven questions inquiring about depression, and seven questions inquiring about anxiety. Higher scores are indicative of more complaints of depression and anxiety respectively. Scores on both subscales range from 0 to 21. The HADS is a valid measure of anxiety and depression in people with MS,^27^ avoiding the use of somatic confounding variables.^2^ A cut-off score of 8 is regarded as indicative of probable depression and/or probable generalized anxiety in people with MS in the respective subscales.^27^

#### Coping style

The Coping Inventory for Stressful Situations (CISS) is a 48-item self-report questionnaire that measures preferred coping styles.^28^ It discriminates between three main coping styles: task-oriented coping, emotion-oriented coping and avoidance-oriented coping. Task-oriented coping is defined as an attempt to solve the problem or alter the situation, while emotion-oriented coping contains the emotional reactions aimed to reduce stress. Avoidance-oriented coping is characterised by activities undertaken in order to avoid stressful situation, e.g. seeking distraction or social diversion.^28^ A higher score on each of the subscales is reflective of more frequent usage of that specific coping style.

#### Work difficulties

To examine MS-related work difficulties the Multiple Sclerosis Work Difficulties Questionnaire-23 (MSWDQ-23) was used.^13^ The MSWDQ-23 consists of three subscales: psychological/cognitive work barriers, physical barriers and external barriers. The subscale scores are computed by summing the observed item scores (ranging from 0 to 4), divided by the total of possible items in the subscale, and then multiplying this score by 100. In this manner, all subscale scores range from 0 to 100. The total score is the average of the three subscale scores. A higher score is reflective of more work difficulties.

#### Demographics and disease characteristics

Regarding demographics, we assessed gender, age and educational level. Educational levels were divided into three categories: lower (completed low-level secondary school), middle and higher education (finished secondary school at medium level and finished secondary school at the highest level, respectively).

In terms of disease characteristics, we assessed disease duration in years using a single item question. We assessed MS-related disability using the Expanded Disability Status Scale (EDSS).^29^ The EDSS scoring was conducted by the neurologist in the outpatient centre. The scores range from 0 to 10, with a higher score being indicative of more disability due to MS.

### Statistical analysis

We created several additive multiple moderation models with hierarchical entry.^30^ First, we ran three regression analyses with anxiety as the independent variable and work difficulties as the dependent variable, using IBM SPSS Version 27. To decrease the chance of multicollinearity, mean centring was applied to all numeric predictors. The first model included only demographics (age, gender, education) and disease characteristics (disease duration and MS-related disability). In the second model anxiety and the three coping styles were added. In the third model, the three interaction effects between anxiety and the three coping styles were added. According to the work by Harrell,^31^ it is advocated to first perform an overall test of the model with the moderator effects together, before looking at separate interactions. Correlation coefficients between the interaction effects were all ≤0.1. Thereafter, ANOVAs were checked for model selection. The same procedure was repeated for depression.

Additionally, we ran several exploratory analyses. Differences in work difficulties were examined between people scoring above and below cut-off scores of the HADS using Mann–Whitney *U* tests. Finally, regression analyses were repeated to explore the effect of depression and anxiety scores above the cut-off score. We created dummy variables for either depression or anxiety with two categories, i.e. scoring below the cut-off score on the HADS (0) and scoring above the cut-off score (1). We added the dummy variable (1) to the regression analyses. Additionally, we repeated the same regressions for people with comorbid anxiety and depression (CAD). We created a dummy variable for people scoring above the cut-off score for both depression and anxiety (1) and added this variable to the regression analyses. Finally, we ran exploratory analyses using the subscales of the MSWDQ-23 as outcome measures. All exploratory analyses will be added to the supplemental materials. Significant interaction effects were probed using the macro PROCESS for SPSS. We considered *p* < 0.05 to be statistically significant.

## Results

### Participants

We included 239 employed participants with MS. Neurological data was missing for 20 participants, resulting in 219 people being included in the analyses. Participant characteristics are presented in Table 1.

**Table 1.** Demographics, disease-related, psychological and occupational characteristics of the participants.

	*N* (%)	Mean (SD)	Median (IQR)
Gender (female)	172 (78.5%)		
Age (years)			43 (25)
Educational level			
Higher	94 (42.9%)		
Middle	86 (39.3%)		
Lower	39 (17.8%)		
MS-related disability (EDSS score)			2.0 (1)
Disease duration (y)			5.3 (8)
Depression score			2.0 (4)
Depression score above cut-off (N)	22 (10%)		
Anxiety score			5.0 (5)
Anxiety score above cut-off (N)	58 (26.5%)		
Comorbid anxiety and depression above cut-off (N)	20 (8.4%)		
Coping styles			
Task-oriented coping			61 (9)
Emotion-oriented coping		37.2 (10.7)	
Avoidance-oriented coping		46.1 (9.3)	
Work difficulties			15.4 (19.3)

*N*  =  219. The values indicate median and interquartile range (IQR) (given the lack of a normal distribution), *N* (%) for depression and anxiety scores above the cut-off, gender and education, or, in case of the normally distributed variables emotion- and avoidance-oriented coping mean and standard deviation [SD]).

#### Regression model with anxiety as predictor of work difficulties

Firstly, Model 1 consisted of age, MS-related disability, gender, education and disease duration. The results of the regression indicated that the model explained 9% of the variance and that the model was significant (*F*_(6,209)_  =  3.62, *p*  =  0.002). MS-related disability was a significant predictor of work difficulties.

Secondly, Model 2 added anxiety and the three coping styles. The equation was significant (*F*_(10,205)_  =  13.14, *p* < 0.001, *R*^2^  =  0.39). Anxiety, avoidance-oriented coping, emotion-oriented coping and MS-related disability were significant predictors (see Table 2).

**Table 2.** Summary of multiple regression analysis with anxiety predicting work difficulties (model 2).

Work difficulties					
Variable	*B*	LB	UB	β	*p* value
Constant	18.59	15.21	21.96		**0**.**001**
Age	−0.05	−0.22	0.12	−0.04	0.561
MS-related disability (EDSS)	2.76	1.60	3.91	0.28	**0**.**001**
Gender male	−0.06	−3.37	3.25	−0.01	0.970
Education middle	−1.53	−5.48	2.43	−0.06	0.448
Education high	−2.24	−6.31	1.82	−0.09	0.278
Disease duration	−0.06	−0.29	0.17	−0.03	0.612
Anxiety	1.51	1.04	1.98	0.422	**0**.**001**
Avoidance-oriented coping	0.16	0.01	0.32	0.12	**0**.**040**
Emotion-oriented coping	0.18	0.03	0.33	0.16	**0**.**017**
Task-oriented coping	0.11	−0.05	0.27	0.08	0.186

LB: lower bound; UB: upper bound. Bold values denote statistical significance at the *p* < 0.05 level.

Finally, in model 3 the interaction effects between anxiety and the three coping styles were added. The equation was significant (*F*_(13,202)_ = 10.01, *p* < 0.001, *R*^2^ = 0.39). Anxiety, avoidance-related coping, emotion-related coping and MS-related disability were significant predictors. None of the interaction effects were significant, indicating no moderation effects of coping (see Table 3).

**Table 3.** Summary of multiple regression analysis with anxiety predicting work difficulties (model 3).

Work difficulties					
Variable	*B*	LB	UB	β	*p* value
Constant	18.45	14.92	21.98		**0**.**001**
Age	−0.05	−0.23	0.12	−0.04	0.558
MS-related disability (EDSS)	2.72	1.56	3.89	0.28	**0**.**001**
Gender male	−0.10	−3.43	3.24	−0.01	0.955
Education middle	−1.58	−5.60	2.44	−0.06	0.440
Education high	−2.22	−6.36	1.92	−0.09	0.291
Disease duration	−0.06	−0.30	0.17	−0.03	0.597
Anxiety	1.48	0.97	1.99	0.41	**0**.**001**
Avoidance-oriented coping	0.16	0.01	0.32	0.12	**0**.**040**
Emotion-oriented coping	0.18	0.03	0.33	0.15	**0**.**021**
Task-oriented coping	0.11	−0.06	0.27	0.08	0.200
Anxiety x avoidance-oriented coping	−0.01	−0.05	0.03	−0.03	0.655
Anxiety x emotion-oriented coping	0.01	−0.03	0.05	0.03	0.642
Anxiety x task-oriented coping	−0.01	−0.05	0.04	−0.01	0.964

LB: lower bound; UB: upper bound. Bold values denote statistical significance at the *p* < 0.05 level.

Thereafter, we looked at the ANOVAs for model selection. Model 2 significantly improved the first model *F_Change_* (4,205)  =  24.92, *p* < 0.001. Adding the interaction effects in Model 3 did not improve the model fit *F_Change_* (3,202)  =  0.15, *p*  =  0.929. Therefore, the second model is considered the best model.

#### Exploratory analyses with anxiety using subscales of the MSWDQ-23 as outcome measures

With respect to the Psychological/Cognitive subscale, the second model was considered the best model (*F*_(10,205)_  =  9.62, *p* < 0.001), *R*^2^ = 0.32. More MS-related disability, anxiety and emotion-oriented coping were significantly related to more Psychological/Cognitive work barriers.

For the Physical subscale, we observed similar outcomes to the total scale. The second model was considered the best model (*F*_(10,205)_  =  13.84, *p* < 0.001), explaining 40% of the variance. Anxiety, MS-related disability, avoidance- and emotion-oriented coping were significantly positively related to Physical work barriers.

Finally, Model 2 was selected as the best model for External barriers (*F*_(10,205)_  =  6.87, *p* < 0.001), *R*^2^  =  0.25. MS-related disability and anxiety were significant positive predictors of External work barriers. Regression models and tables using subscales of the MSWDQ-23 as outcome measures are reported in supplemental material.

#### Regression model with depression as predictor of work difficulties

Model 1 is identical to Model 1 examining anxiety. Thereafter, we added depression and the three coping styles in Model 2. The equation was significant (*F*_(10,205)_  =  14.98, *p* < 0.001, *R*^2^  =  0.42), and depression, avoidance-related coping, emotion-oriented coping and MS-related disability were significant predictors (see Table 4).

**Table 4.** Summary of multiple regression analysis with depression predicting work difficulties (model 2).

Work difficulties					
Variable	*B*	LB	UB	β	*p* value
Constant	19.34	16.08	22.60		**0**.**001**
Age	−0.03	−0.20	0.14	−0.02	0.721
MS-related disability (EDSS)	2.29	1.14	3.43	0.23	**0**.**001**
Gender male	−0.63	−3.86	2.61	−0.02	0.703
Education middle	−2.75	−6.56	1.06	−0.11	0.156
Education high	−2.10	−6.06	1.85	−0.08	0.296
Disease duration	−0.06	−0.28	0.17	−0.03	0.618
Depression	1.82	1.33	2.31	0.43	**0**.**001**
Avoidance-oriented coping	0.23	0.08	0.38	0.17	**0**.**003**
Emotion-oriented coping	0.28	0.15	0.41	0.24	**0**.**001**
Task-oriented coping	0.09	−0.07	0.246	0.07	0.252

LB: lower bound; UB: upper bound. Bold values denote statistical significance at the *p* < 0.05 level.

Finally, in model 3, the interaction effects between depression and the three coping styles were included. The equation was significant (*F*_(13,202) _ =  11.51, *p* < 0.001. *R*^2^ = 0.43). Depression, avoidance-oriented coping, emotion-oriented coping and MS-related disability were significant predictors. None of the interaction effects were significant, indicating no moderation effects of coping (see Table 5).

**Table 5.** Summary of multiple regression analysis with depression predicting work difficulties (Model 3).

Work difficulties					
Variable	*B*	LB	UB	β	*p* value
Constant	19.23	15.85	22.61		**0**.**001**
Age	−0.02	−0.19	0.15	−0.02	0.820
MS-related disability (EDSS)	2.32	1.16	3.48	0.24	**0**.**001**
Gender male	−0.78	−4.07	2.50	−0.03	0.639
Education middle	−2.43	−6.34	1.47	−0.10	0.221
Education high	−1.90	−5.95	2.15	−0.08	0.357
Disease duration	−0.05	−0.28	0.17	−0.03	0.643
Depression	1.89	1.45	2.43	0.45	**0**.**001**
Avoidance-oriented coping	0.23	0.07	0.38	0.17	**0**.**004**
Emotion-oriented coping	0.28	0.15	0.41	0.24	**0**.**001**
Task-oriented coping	0.10	−0.06	0.26	0.08	0.200
Depression x avoidance-oriented coping	0.02	−0.02	0.06	0.06	0.347
Depression x emotion-oriented coping	0.00	−0.04	0.04	0.01	0.993
Depression x task-oriented coping	0.01	−0.05	0.07	0.02	0.699

LB: lower bound; UB: upper bound. Bold values denote statistical significance at the *p* < 0.05 level.

We looked at the ANOVAs for model selection. Adding the variables of Model 2 significantly improved the first model (*F_Change_*(4,205)  =  29.10, *p* < 0.001). Adding the interaction effects in Model 3 did not improve the model fit (*F_Change_* (3,202)  =  0.37, *p* = 0.772). Consequently, we selected the second model as the best model.

#### Exploratory analyses with depression using subscales of the MSWDQ-23 as outcome measures

Firstly, we fitted models for the Psychological/Cognitive subscale. Model 2 was considered the best model *F*_(10,205)_  =  9.96, *p* < 0.001, *R*^2^  =  0.33). Depression, avoidance-oriented coping and emotion-oriented coping were significantly positively associated with Psychological/Cognitive work barriers.

For the Physical subscale, we selected the second model as the best model (*F*_(10,205)_  =  13.70, *p* < 0.001), explaining 40% of the variance. More depression, MS-related disability, avoidance- and emotion-oriented coping were significantly related to more Physical work barriers.

Finally, Model 2 was considered the best model for External barriers (*F*_(10,205)_  =  9.09, *p* < 0.001), *R*^2^  =  0.31. Depression, MS-related disability, avoidance- and emotion-oriented coping were significantly positively related to External work barriers. Regression models and tables using subscales of the MSWDQ-23 as outcome measures are reported in supplemental material.

### Dichotomised anxiety and depression scores as predictors of work difficulties

A Mann–Whitney *U* test indicated that people scoring above the cut-off score for depression reported more work difficulties (*Mdn*  =  29.6) than people who did not (*Mdn*  =  13.6, *U*  =  1286.5, *p* < 0.001). Similarly, people scoring above the cut-off score for anxiety reported more work difficulties (*Mdn*  =  28.1) than people who did not (*Mdn*  =  10.9, *U* = 2126.5, *p* < 0.001).

Conducting regression analyses using the dichotomised depression and anxiety variables (HADS-score being above the cut-off) resulted in similar patterns to the analyses with continuous variables. Regarding anxiety, the second model was considered the best model *F*_(10,205)_  =  12.93, *p* < 0.001 with *R*^2^  =  0.39. Anxiety, avoidance-oriented coping, emotion-oriented coping and MS-related disability were significantly associated with work difficulties. With regard to depression, the second model was considered the best model too *F*_(10,205)_  =  9.74, *p* < 0.001 with *R*^2^  =  0.32. Depression, avoidance-oriented coping, emotion-oriented coping and MS-related disability were significant predictors of work difficulties. Finally, regression models were run with CAD and resulted in similar outcomes. Details of the regression analyses are included in the online supplementary material.

## Discussion

The current study aimed to examine the relationships between anxiety, depression, coping and work difficulties in people with MS. The results show that anxiety, emotion-oriented coping, avoidance-oriented coping and MS-related disability were positively associated with work difficulties. Additionally, we observed a similar pattern for depression: more depressive symptoms, emotion-oriented coping, avoidance-oriented coping and MS-related disability were significantly related to more work difficulties. In both models, the interaction effects did not reach significance, and none of the demographic characteristics were significantly related to work difficulties. Similar results were found when dichotomised depression and anxiety scores were added to the regression analyses. Similarly, we observed identical results for people suffering from CAD.

These findings highlight the importance of mental health in the workplace. In line with previous studies,^13,15^ this study shows that symptoms of depression are important contributors to occupational difficulties. In addition, we found that anxiety was related to work difficulties as well. Although people scoring above the cut-off score for depression and anxiety report more work difficulties than people scoring below the cut-off score, the same predictors for work difficulties were observed in the analyses including scores above the cut-off.

In a clinical setting, cognitive behavioural therapy (CBT) is an established intervention to reduce both depression and anxiety in MS.^32,33^ Core components of CBT might also be useful in a work setting. A Norwegian randomised control trial^34^ implemented a work intervention with work-focused CBT and individual job support in a sample of people with common mental disorders. Their analyses yielded twofold results. Firstly, they showed that significantly more people in the intervention group were able to increase or maintain work participation when compared to the control group. This effect remained significant even at a follow-up after 18 months. Moreover, the programme was able to alleviate symptoms of depression and anxiety more than care as usual.^34^ Future research is needed on the application and efficacy of work-focused CBT in workers with MS.

In accordance with previous work, both anxiety and depression are positively related to work difficulties. However, other studies have shown a differential relationship in that being employed was related to higher depression scores.^7^ The authors hypothesised that the demands of maintaining employment are a source of stress and may cause elevated levels of depression in people with MS. These divergent findings may indicate a reciprocal relationship between occupational functioning and mental health, in which mental health influences occupational functioning as well as the other way around. Subsequent (longitudinal) research is needed to unravel the complex relationship between occupational functioning and mental health in MS.

Rather than moderation effects, we observed direct effects of coping on work difficulties. Specifically, the usage of emotion-oriented coping and avoidance-oriented coping was related to more work difficulties. Coping can be conceptualised as a ‘constantly changing cognitive and behavioural effort to manage specific external or internal demands that are appraised as taxing’.^12^ This definition considers coping to be an ongoing evaluation process and recognises that the effectiveness of a particular coping style is context-dependent. Although avoidant- and emotion-oriented strategies are often considered maladaptive, in specific situations denial can offer an individual time to adjust to a changing situation, while emotion-focused strategies can alleviate stress and anxiety in situations that are perceived beyond the control of the individual.^12^

However, in a work setting avoidance-oriented and emotion-oriented coping styles are often associated with worse work outcomes such as (shorter time to) unemployment and negative work events in people with MS,^18,19,35^ and worse work ability in healthy workers.^22^ Research (in people without MS) indicates that strategies reflecting disengagement coping with stress, such as avoidance and passive reaction patterns, and expressing emotions are associated with less perceived control. This may cause people using these coping styles to experience a lack of possibilities to confront the stressor.^36^ In the workplace, this might prevent workers with MS from tackling work difficulties and gaining the proper accommodations.

Although previous studies have linked more task-oriented coping to employment,^4^ the current study did not find a significant association between task-oriented coping and work difficulties. This unexpected finding might be partly explained by earlier work^35^ in which the relationship between coping styles and negative work events and accommodations was examined. The results indicated that people who employed more task-oriented coping more frequently reported using flexible scheduling as an accommodation. Task- or problem-oriented coping increases the perceived control over the situation and hereby the drive to alter the situation.^12^ Specifically, one might anticipate possible work difficulties and take the right steps to prevent them from happening. So rather than directly affecting work difficulties, task-oriented coping might prevent work difficulties from happening through a different underlying mechanism.

Interestingly, both models show a similar pattern of significant predictors. This is not unexpected, given the common co-occurrence of depression and anxiety in people with MS.^2^ Studies suggest that the presence of anxiety might be a predictor of depression in that anxiety can induce negative emotions which can lead to depressive symptoms.^37^ However, other studies suggest that depression and anxiety each may have a differential impact on employment.^38^ Therefore, examining the separate contributions of depression and anxiety to employment remains relevant.

Additionally, our results suggest that MS-related disability is related to work difficulties. This finding is in line with a multitude of studies on occupational functioning in MS where MS-related disability, i.e. EDSS and occupational functioning are interconnected.^3^

Finally, we performed exploratory analyses using the subscales of the MSWDQ-23 as outcome measures (as reported in supplemental material). Although the majority of the findings was similar to the general findings, we observed a few differences. For instance, although coping seems to be a relevant factor in work difficulties, neither of the coping styles reached significance with respect to external work barriers in the model with anxiety. As for the model including depression, both avoidance- and emotion-oriented coping were related to external barriers. This difference is unexpected, and more research is needed to identify specific profiles at risk for particular work difficulties.

## Strengths and limitations

A strength of the current study is the relatively large sample of working people with MS (*N*  =  219).

Several limitations should be kept in mind when interpreting the results. Firstly, the current sample consists of people with MS in paid employment, with relatively limited disability (EDSS median  =  2.0, IQR  =  1), and relatively low HADS-scores (Depression median  =  2.0, IQR  =  4, Anxiety median  =  5.0, IQR  =  5). Additionally, people with a DSM-5 diagnosis of a mood disorder were excluded from participating. This may lead to an undervaluation of the effect that more severe mood complaints may have on work difficulties. Hence, caution is warranted in the generalisation of the results. Secondly, due to the cross-sectional design, causal inferences cannot be drawn. Mental health problems and work difficulties may have a reciprocal relationship.^7^

Finally, for the current study, we have focused on isolated mechanisms of mental health and occupational functioning. However, occupational functioning is a multifactorial concept, and the impact of clinical characteristics and work environment needs to be taken into account in future studies.^12^ Symptoms such as fatigue have a known impact on occupational functioning,^3^ as well as an association with mental health in people with MS.^2^

## Conclusions

Depression, anxiety and MS-related disability were positively related to work difficulties in people with MS with limited disability. There were no moderation effects of coping styles, but emotion-oriented coping and avoidance-oriented coping directly affected work difficulties in that more frequent usage of these particular coping styles was related to more work difficulties. Future research should examine the application of work-based interventions such as work-focused CBT.

## Supplemental Material

sj-docx-1-mso-10.1177_20552173221116282 - Supplemental material for Work difficulties in people with multiple sclerosis: The role of anxiety, depression and coping Click here for additional data file. Supplemental material, sj-docx-1-mso-10.1177_20552173221116282 for Work difficulties in people with multiple sclerosis: The role of anxiety, depression and coping by EEA van Egmond, K van der Hiele, DAM van Gorp, PJ Jongen, JJL van der Klink, MF Reneman, EAC Beenakker, JJJ van Eijk, STFM Frequin, K de Gans, BM van Geel, OHH Gerlach, GJD Hengstman, JP Mostert, WIM Verhagen, HAM Middelkoop and LH Visser in Multiple Sclerosis Journal – Experimental, Translational and Clinical

## References

[1] Boeschoten RE, Braamse AMJ, Beekman ATF, et al. Prevalence of depression and anxiety in multiple sclerosis: a systematic review and meta-analysis. J Neurol Sci 2017; 372: 331–341. 28017241 10.1016/j.jns.2016.11.067

[2] Feinstein A, Magalhaes S, Richard J, Audet B and Moore C. The link between multiple sclerosis and depression. Nat Rev Neurol 2014; 10: 507–517. 25112509 10.1038/nrneurol.2014.139

[3] Raggi A, Covelli V, Schiavolin S, Scaratti C, Leonardi M and Willems M. Work-related problems in multiple sclerosis: a literature review on its associates and determinants. Disabil Rehabil 2016; 38: 936–944. 26213244 10.3109/09638288.2015.1070295

[4] Dorstyn DS, Roberts RM, Murphy G and Haub R. Employment and multiple sclerosis: a meta-analytic review of psychological correlates. J Health Psychol 2019; 24: 38–51. 28810436 10.1177/1359105317691587

[5] Honan CA Brown RF Batchelor J . Perceived cognitive difficulties and cognitive test performance as predictors of employment outcomes in people with multiple sclerosis. J Int Neuropsychol Soc 2015; 21: 156–168. 25727930 10.1017/S1355617715000053

[6] Strober LB Chiaravalloti N DeLuca J . Should I stay or should I go? A prospective investigation examining individual factors impacting employment status among individuals with multiple sclerosis (MS). Work 2018; 59: 39–47. 29355118 10.3233/WOR-172667 PMC13528306

[7] Smith MM Arnett PA . Factors related to employment status changes in individuals with multiple sclerosis. Mult Scler 2005; 11: 602–609. 16193900 10.1191/1352458505ms1204oa

[8] Cadden M Arnett P . Factors associated with employment Status in individuals with multiple sclerosis. Int J MS Care 2015; 17: 284–291. 26664334 10.7224/1537-2073.2014-057 PMC4673921

[9] D'Hooghe MB, De Cock A, Benedict RHB, et al. Perceived neuropsychological impairment inversely related to self-reported health and employment in multiple sclerosis. Eur J Neurol 2019; 26: 1447–1454. 31183915 10.1111/ene.14012

[10] Ernstsson O, Gyllensten H, Alexanderson K, Tinghög P, Friberg E and Norlund A. Cost of illness of multiple sclerosis – a systematic review. PLoS One 2016; 11: e0159129. 27411042 10.1371/journal.pone.0159129 PMC4943600

[11] Chen J, Taylor B, Palmer AJ, et al. Estimating MS-related work productivity loss and factors associated with work productivity loss in a representative Australian sample of people with multiple sclerosis. Mult Scler 2019; 25: 994–1004. 29911469 10.1177/1352458518781971

[12] Vijayasingham L Mairami FF . Employment of patients with multiple sclerosis: the influence of psychosocial-structural coping and context. Degener Neurol Neuromuscul Dis 2018; 8: 15–24. 30050385 10.2147/DNND.S131729 PMC6053901

[13] Honan CA Brown RF Hine DW . The Multiple Sclerosis Work Difficulties Questionnaire (MSWDQ): development of a shortened scale. Disabil Rehabil 2014; 36: 635–641. 23786346 10.3109/09638288.2013.805258

[14] Honan CA, Brown RF, Hine DW, et al. The multiple sclerosis work difficulties questionnaire. Mult Scler 2012; 18: 871–880. 22146603 10.1177/1352458511431724

[15] van Egmond E, van Gorp D, Honan C, et al. A Dutch validation study of the multiple sclerosis work difficulties questionnaire in relapsing remitting multiple sclerosis. Disabil Rehabil 2021; 43: 1924–1933. 31702954 10.1080/09638288.2019.1686072

[16] van der Hiele K, van Gorp DAM, van Egmond EEA, et al. Self-reported occupational functioning in persons with relapsing-remitting multiple sclerosis: does personality matter? J Neurol Sci 2021; 427: 117561. 34216973 10.1016/j.jns.2021.117561

[17] Hartoonian N,Terrill AL, Beier ML, Turner AP, Day MA and Alschuler KN. Predictors of anxiety in multiple sclerosis. Rehabil Psychol 2015; 60: 91–98. 25496434 10.1037/rep0000019 PMC4339307

[18] Grytten N, Skår AB, Aarseth JH, et al. The influence of coping styles on long-term employment in multiple sclerosis: a prospective study. Mult Scler 2017; 23: 1008–1017. 27600114 10.1177/1352458516667240

[19] Strober LB Arnett PA . Unemployment among women with multiple sclerosis: the role of coping and perceived stress and support in the workplace. Psychol Health Med 2016; 21: 496–504. 26456395 10.1080/13548506.2015.1093645

[20] Holland DP, Schlüter DK, Young CA, et al. Use of coping strategies in multiple sclerosis: association with demographic and disease-related characteristics. Mult Scler Relat Disord 2019; 27: 214–222. 30412819 10.1016/j.msard.2018.10.016

[21] Lode K, Bru E, Klevan G, Myhr KM, Nyland H and Larsen JP. Coping with multiple sclerosis: a 5-year follow-up study. Acta Neurol Scand 2010; 122: 336–342. 20047563 10.1111/j.1600-0404.2009.01313.x

[22] van de Vijfeijke H, Leijten FRM, Ybema JF, et al. Differential effects of mental and physical health and coping style on work ability: a 1-year follow-up study among aging workers. J Occup Environ Med 2013; 55: 1238–1243. 24064781 10.1097/JOM.0b013e3182a2a5e1

[23] van der Hiele K, van Gorp DAM, Heerings MAP, et al. The MS@Work study: a 3-year prospective observational study on factors involved with work participation in patients with relapsing-remitting Multiple Sclerosis. BMC Neurol 2015; 15: 134. 26264389 10.1186/s12883-015-0375-4 PMC4531500

[24] Polman CH, Reingold SC, Banwell B, et al. Diagnostic criteria for multiple sclerosis: 2010 revisions to the McDonald criteria. Ann Neurol 2011; 69: 292–302. 21387374 10.1002/ana.22366 PMC3084507

[25] American Psychiatric Association . Diagnostic and statistical manual of mental disorders. 5th ed. Washington: American Psychiatric Publishing Inc, 2013.

[26] Spinhoven P, Ormel J, Sloekers PP, Kempen GI, Speckens AE and van Hemert AM. A validation study of the Hospital Anxiety and Depression Scale (HADS) in different groups of Dutch subjects. Psychol Med 1997; 27: 363–370. 9089829 10.1017/s0033291796004382

[27] Honarmand K Feinstein A . Validation of the hospital anxiety and depression scale for use with multiple sclerosis patients. Mult Scler 2009; 15: 1518–1524. 19965520 10.1177/1352458509347150

[28] Endler N Parker JD . Coping inventory for stressful situations (CISS): manual multi-health systems. Toronto, Canada: Multi-Health Systems, 1999.

[29] Kurtzke JF . Rating neurologic impairment in multiple sclerosis: an expanded disability status scale (EDSS). Neurology 1983; 33: 1444–1452. 6685237 10.1212/wnl.33.11.1444

[30] Hayes AF . Introduction to mediation, moderation, and conditional process analysis: a regression-based approach. 2nd ed. New York: Guilford Press, 2018.

[31] Harrell FEJ . Regression modeling strategies. New York: Springer, 1999.

[32] Pahlavanzadeh S Abbasi S Alimohammadi N . The effect of group cognitive behavioral therapy on stress, anxiety, and depression of women with multiple sclerosis. Iran J Nurs Midwifery Res 2017; 22: 271–275. 28904538 10.4103/1735-9066.212987 PMC5590355

[33] Hind D, Cotter J, Thake A, et al. Cognitive behavioural therapy for the treatment of depression in people with multiple sclerosis: a systematic review and meta-analysis. BMC Psychiatry 2014; 14: 5. 24406031 10.1186/1471-244X-14-5 PMC3890565

[34] Reme SE, Grasdal AL, Løvvik C, Lie SA and Øverland S. Work-focused cognitive-behavioural therapy and individual job support to increase work participation in common mental disorders: a randomised controlled multicentre trial. Occup Environ Med 2015; 72: 745–752. 26251065 10.1136/oemed-2014-102700 PMC4602235

[35] van der Hiele K, van Gorp DAM, Benedict RHB, et al. Coping strategies in relation to negative work events and accommodations in employed multiple sclerosis patients. Mult Scler J Exp Transl Clin 2016; 2: 2055217316680638. 28607745 10.1177/2055217316680638 PMC5408754

[36] Dijkstra MT Homan AC . Engaging in rather than disengaging from stress: effective coping and perceived control. Front Psychol 2016; 7: 1415. 27708603 10.3389/fpsyg.2016.01415 PMC5030286

[37] Gay MC, Bungener C, Thomas S, et al. Anxiety, emotional processing and depression in people with multiple sclerosis. BMC Neurol 2017; 17: 43. 28231828 10.1186/s12883-017-0803-8 PMC5324294

[38] Gill S, Santo J, Blair M and Morrow SA. Depressive symptoms are associated with more negative functional outcomes than anxiety symptoms in persons with multiple sclerosis. J Neuropsychiatry Clin Neurosci 2019; 31: 37–42. 30187820 10.1176/appi.neuropsych.18010011

