# Supplementary material for: Work difficulties in people with multiple sclerosis: The role of anxiety, depression and coping
Source: Mult Scler J Exp Transl Clin. 2022 Sep 4;8(3):20552173221116282. doi: 10.1177/20552173221116282 (PMC9445483; doi:10.1177/20552173221116282)
Supplement: sj-docx-1-mso-10.1177_20552173221116282 - Supplemental material for Work difficulties in people with multiple sclerosis: The role of anxiety, depression and coping [file sj-docx-1-mso-10.1177_20552173221116282.docx]

**Supplemental material including exploratory analyses**

**Anxiety**

Firstly, we ran model 1 consisting of MS-related disability, gender, age, disease duration and education. This resulted in a significant equation with *F*(6,209)=3.62, *p*=0.002, *R*^2^=0.09. MS-related disability was significantly associated with work difficulties.

Secondly, we added anxiety (dichotomised, as a dummy variable) and the coping styles in model 2. The equation was significant with *F*(10,205)=12.93, *p*<0.001 with *R*^2^=0.39. Anxiety, avoidance-oriented coping, emotion-oriented coping and MS-related disability were significant predictors. Model 2 is summarised in Supplementary Table 1.

Supplementary Table 1. Summary of multiple regression analysis predicting work difficulties using a dichotomised measure of anxiety (Model 2)

| Work difficulties |  |  |  |  |  |
| --- | --- | --- | --- | --- | --- |
| Variable | B | LB | UB | β | *p*-value |
| Constant | 16.51 | 12.97 | 20.10 |  | **0.001** |
| Age | -0.11 | -0.28 | 0.06 | -0.08 | 0.203 |
| MS-related disability (EDSS) | 2.92 | 1.77 | 4.07 | 0.30 | **0.001** |
| Gender male | 0.51 | -2.81 | 3.82 | 0.02 | 0.764 |
| Education middle | -2.46 | -6.39 | 1.47 | -0.10 | 0.218 |
| Education high | -3.32 | -7.36 | 0.73 | -0.13 | 0.107 |
| Disease duration | -0.11 | -0.34 | 0.12 | -0.06 | 0.364 |
| Anxiety | 10.53 | 7.18 | 13.87 | 0.38 | **0.001** |
| Avoidance-oriented coping | 0.18 | 0.03 | 0.33 | 0.13 | **0.023** |
| Emotion-oriented coping | 0.26 | 0.13 | 0.40 | 0.23 | **0.001** |
| Task-oriented coping | 0.13 | -0.04 | 0.29 | 0.09 | 0.126 |

Thirdly, the interaction effects were added in Model 3. This yielded a significant equation F(13,202)=9.89, p<0.001 with R^2^=0.39. Anxiety, emotion-oriented coping and MS-related disability were significantly related to work difficulties. None of the interaction effects were significant, demonstrating there is no moderation effect of coping style. Model 3 is depicted in Supplementary Table 2.

Supplementary table 2. Summary of multiple regression analysis predicting work difficulties using a dichotomised measure of anxiety (Model 3)

| Work difficulties |  |  |  |  |  |
| --- | --- | --- | --- | --- | --- |
| Variable | B | LB | UB | β | *p*-value |
| Constant | 16.35 | 12.74 | 19.96 |  | **0.001** |
| Age | -0.11 | -0.28 | 0.064 | -0.08 | 0.219 |
| MS-related disability (EDSS) | 2.90 | 1.73 | 4.07 | 0.30 | **0.001** |
| Gender male | 0.41 | -2.95 | 3.78 | 0.01 | 0.809 |
| Education middle | -2.38 | -6.41 | 1.64 | -0.09 | 0.244 |
| Education high | -3.19 | -7.29 | 0.91 | -0.13 | 0.126 |
| Disease duration | -0.12 | -0.35 | 0.12 | -0.06 | 0.333 |
| Anxiety | 9.94 | 6.29 | 13.60 | 0.36 | **0.001** |
| Avoidance-oriented coping | 0.17 | -0.01 | 0.35 | 0.13 | 0.062 |
| Emotion-oriented coping | 0.23 | 0.06 | 0.39 | 0.20 | **0.007** |
| Task-oriented coping | 0.13 | -0.05 | 0.31 | 0.09 | 0.162 |
| Anxiety x avoidance oriented coping | 0.03 | -0.33 | 0.38 | 0.01 | 0.886 |
| Anxiety x emotion-oriented coping | 0.13 | -0.18 | 0.44 | 0.06 | 0.411 |
| Anxiety x task-oriented coping | -0.01 | -0.41 | 0.40 | -0.01 | 0.965 |

We looked at the ANOVA’s for model selection. Model 2 significantly improved Model 1 (F_Change_ (4,205)=24.54, p<0.001). Adding the interaction effects in Model 3 did not improve model fit (F_Change_ (3,202)=0.24, *p*=0.87). Consequently, model 2 was selected as the best model.

**Depression**

Firstly, we performed a regression analysis with model 1, identical to the previous Model 1.

Secondly, we included depression (dichotomised, as a dummy variable) and the coping styles in model 2. This resulted in a significant equation with *F*(10,205)=9.74, *p*<0.001 with *R*^2^=0.32. Depression, avoidance-oriented coping, emotion-oriented coping and MS-related disability were significant predictors. Model 2 is described in Supplementary Table 3.

Supplementary Table 3. Summary of multiple regression analysis predicting work difficulties using a dichotomised measure of depression (Model 2)

| Work difficulties |  |  |  |  |  |
| --- | --- | --- | --- | --- | --- |
| Variable | B | LB | UB | β | *p*-value |
| Constant | 18.73 | 15.13 | 22.32 |  | **0.001** |
| Age | -0.11 | -0.29 | 0.07 | -0.08 | 0.238 |
| MS-related disability (EDSS) | 3.06 | 1.85 | 4.27 | 0.31 | **0.001** |
| Gender male | 0.46 | -3.03 | 3.95 | 0.02 | 0.797 |
| Education middle | -3.43 | -7.55 | 0.68 | -0.14 | 0.102 |
| Education high | -3.28 | -7.54 | 0.99 | -0.13 | 0.131 |
| Disease duration | -0.08 | -0.32 | 0.17 | -0.04 | 0.545 |
| Depression | 9.65 | 4.79 | 14.51 | 0.24 | **0.001** |
| Avoidance-oriented coping | 0.20 | 0.04 | 0.37 | 0.15 | **0.015** |
| Emotion-oriented coping | 0.38 | 0.25 | 0.52 | 0.33 | **0.001** |
| Task-oriented coping | 0.09 | -0.08 | 0.25 | 0.06 | 0.321 |

Thirdly, we added the interaction effects in Model 3. The equation was significant F(13,202)=7.80, p<0.001, R^2^=0.33. Depression, avoidance-oriented and emotion-oriented coping and MS-related disability were significantly related to work difficulties. None of the interaction effects were significant, demonstrating there is no moderation effect of coping style. Model 3 is summarised in Supplementary Table 4.

Supplementary table 4. Summary of multiple regression analysis predicting work difficulties using a dichotomised measure of depression (Model 3)

| Work difficulties |  |  |  |  |  |
| --- | --- | --- | --- | --- | --- |
| Variable | B | LB | UB | β | *p*-value |
| Constant | 19.09 | 15.44 | 22.75 |  | **0.001** |
| Age | -0.12 | -0.30 | 0.06 | -0.09 | 0.194 |
| MS-related disability (EDSS) | 2.93 | 1.71 | 4.16 | 0.30 | **0.001** |
| Gender male | 0.54 | -2.98 | 4.06 | 0.02 | 0.761 |
| Education middle | -4.03 | -8.22 | 0.17 | -0.16 | 0.060 |
| Education high | -3.64 | -7.95 | 0.67 | -0.15 | 0.097 |
| Disease duration | -0.07 | -0.31 | 0.18 | -0.04 | 0.595 |
| Depression | 8.22 | 2.00 | 14.44 | 0.20 | **0.010** |
| Avoidance-oriented coping | 0.22 | 0.05 | 0.40 | 0.17 | **0.013** |
| Emotion-oriented coping | 0.38 | 0.24 | 0.53 | 0.33 | **0.001** |
| Task-oriented coping | 0.12 | -0.06 | 0.29 | 0.09 | 0.193 |
| Depression x avoidance oriented coping | -0.28 | -0.82 | 0.26 | -0.07 | 0.303 |
| Depression x emotion-oriented coping | 0.08 | -0.45 | 0.62 | 0.03 | 0.758 |
| Depression x task-oriented coping | -0.49 | -1.10 | 0.11 | -0.10 | 0.109 |

Finally, ANOVA’s were inspected for model selection. Model 2 significantly improved Model 1 (F_Change_ (4,205)=17.24, p<0.001). Adding the interaction effects in Model 3 did not improve model fit (F_Change_ (3,202)=1.21, *p*=0.307). Hence, model 2 was selected as the best model.

**Comorbid anxiety and depression**

Firstly, Model 1 was identical to the first models of the previous analyses.

Secondly, we included comorbid anxiety and depression (CAD) (dichotomised, as a dummy variable) and the coping styles in model 2. This equation was significant with *F*(10,205)=10.09, *p*<0.001 with *R*^2^=0.33. CAD, avoidance-oriented coping, emotion-oriented coping and MS-related disability were significant predictors. Model 2 is described in Supplementary Table 5.

Supplementary Table 5. Summary of multiple regression analysis predicting work difficulties using a dichotomised measure of comorbid anxiety and depression (CAD) (Model 2)

| Work difficulties |  |  |  |  |  |
| --- | --- | --- | --- | --- | --- |
| Variable | B | LB | UB | β | *p*-value |
| Constant | 18.92 | 15.37 | 22.47 |  | **0.001** |
| Age | -.10 | -0.28 | 0.08 | -0.08 | 0.260 |
| MS-related disability (EDSS) | 3.01 | 1.80 | 4.21 | 0.31 | **0.001** |
| Gender male | 0.58 | -2.90 | 4.05 | 0.02 | 0.743 |
| Education middle | -3.68 | -7.77 | 0.41 | -0.15 | 0.078 |
| Education high | -3.55 | -7.78 | 0.68 | -0.14 | 0.100 |
| Disease duration |  |  |  |  |  |
| CAD | 11.10 | 5.92 | 16.27 | 0.25 | **0.001** |
| Avoidance-oriented coping | 0.19 | 0.03 | 0.36 | 0.15 | **0.018** |
| Emotion-oriented coping | 0.37 | 0.23 | 0.51 | 0.32 | **0.001** |
| Task-oriented coping | 0.11 | -0.06 | 0.28 | 0.08 | 0.21 |

Thirdly, we added the interaction effects in Model 3. The equation was significant F(13,202)=7.99, p<0.001, R^2^=0.34. CAD, avoidance-oriented and emotion-oriented coping, MS-related disability and a middle educational level were significantly related to work difficulties. None of the interaction effects were significant, demonstrating there is no moderation effect of coping style. Model 3 is summarised in Supplementary Table 6.

Supplementary Table 6. Summary of multiple regression analysis predicting work difficulties using a dichotomised measure of comorbid CAD (Model 3)

| Work difficulties |  |  |  |  |  |
| --- | --- | --- | --- | --- | --- |
| Variable | B | LB | UB | β | *p*-value |
| Constant | 19.34 | 15.72 | 22.97 |  | **0.001** |
| Age | -0.12 | -0.30 | 0.06 | -0.09 | 0.187 |
| MS-related disability (EDSS) | 2.94 | 1.73 | 4.16 | 0.30 | **0.001** |
| Gender male | 0.71 | -2.79 | 4.21 | 0.02 | 0.690 |
| Education middle | -4.33 | -8.53 | -0.34 | -0.17 | **0.043** |
| Education high | -3.99 | -8.29 | 0.31 | -0.16 | 0.069 |
| Disease duration | -0.08 | -0.32 | 0.16 | -0.04 | 0.521 |
| CAD | 9.38 | 1.70 | 17.06 | 0.21 | **0.017** |
| Avoidance-oriented coping | 0.22 | 0.05 | 0.39 | 0.16 | **0.013** |
| Emotion-oriented coping | 0.38 | 0.24 | 0.52 | 0.33 | **0.001** |
| Task-oriented coping | 0.13 | -0.05 | 0.30 | 0.09 | 0.149 |
| CAD x avoidance oriented coping | -0.26 | -0.80 | 0.29 | -0.07 | 0.348 |
| CAD x emotion-oriented coping | 0.03 | -0.57 | 0.62 | 0.01 | 0.924 |
| CAD x task-oriented coping | -0.51 | -1.25 | 0.24 | -0.09 | 0.183 |

Subsequently, ANOVA’s were inspected for model selection. Model 2 significantly improved Model 1 (F_Change_ (4,205)=18.02, *p*<0.001). Adding the interaction effects in Model 3 did not improve model fit (F_Change_ (3,202)=0.98, *p*=0.402). Hence, model 2 was selected as the best model.

**Exploratory analyses using subscales of the MSWDQ-23 as outcome measures**

*Psychological/cognitive work barriers: Anxiety*

Model 2 was selected as the best model (Supplementary Table 7), (*F*(10,205)=9.62 *p*<0.001), explaining 32% of the variance. MS-related disability, anxiety and emotion-oriented coping were significantly related to psychological/cognitive work barriers.

Supplementary Table 7. Summary of multiple regression analysis with anxiety predicting psychological/cognitive work barriers (Model 2)

| Psychological/cognitive work barriers |  |  |  |  |  |
| --- | --- | --- | --- | --- | --- |
| Variable | B | LB | UB | β | *p*-value |
| Constant | 18.30 | 14.43 | 22.18 |  | **0.001** |
| Age | -0.06 | -0.25 | 0.14 | -0.04 | 0.572 |
| MS-related disability (EDSS) | 1.49 | 0.16 | 2.81 | 0.14 | **0.028** |
| Gender male | -0.36 | -4.16 | 3.44 | -0.01 | 0.853 |
| Education middle | -2.02 | -6.56 | 2.52 | -0.07 | 0.382 |
| Education high | -0.96 | -5.63 | 3.71 | -0.04 | 0.686 |
| Disease duration | 0.01 | -0.26 | 0.27 | 0.01 | 0.968 |
| Anxiety | 1.59 | 1.05 | 2.13 | 0.41 | **0.001** |
| Avoidance-oriented coping | 0.13 | -0.05 | 0.31 | 0.09 | 0.145 |
| Emotion-oriented coping | 0.22 | 0.05 | 0.39 | 0.17 | **0.012** |
| Task-oriented coping | 0.08 | -0.11 | 0.26 | 0.05 | 0.418 |

*Psychological/cognitive work barriers: Depression*

The best model was model 2 (Supplementary Table 8; *F*(10,205)=9.96, *p*<0.001, *R*^2^=0.33). Depression, avoidance-oriented coping and emotion-oriented coping were significantly related to psychological/cognitive work barriers.

Supplementary Table 8. Summary of multiple regression analysis with depression predicting psychological/cognitive work barriers (Model 2)

| Psychological/cognitive work barriers |  |  |  |  |  |
| --- | --- | --- | --- | --- | --- |
| Variable | B | LB | UB | β | *p*-value |
| Constant | 19.16 | 15.34 | 22.99 |  | **0.001** |
| Age | -0.04 | -0.24 | 0.15 | -0.03 | 0.660 |
| MS-related disability (EDSS) | 1.07 | -0.27 | 2.41 | 0.10 | 0.116 |
| Gender male | -0.87 | -4.65 | 2.92 | -0.03 | 0.653 |
| Education middle | -3.38 | -7.84 | 1.08 | -0.12 | 0.137 |
| Education high | -0.98 | -5.62 | 3.66 | -0.04 | 0.677 |
| Disease duration | 0.01 | -0.26 | 0.27 | 0.01 | 0.987 |
| Depression | 1.76 | 1.18 | 2.33 | 0.38 | **0.001** |
| Avoidance-oriented coping | 0.20 | 0.02 | 0.37 | 0.14 | **0.028** |
| Emotion-oriented coping | 0.34 | 0.19 | 0.49 | 0.27 | **0.001** |
| Task-oriented coping | 0.06 | -0.13 | 0.24 | 0.04 | 0.536 |

*Physical work barriers: Anxiety*

The models and significant predictors were identical to the models with the total score as the dependent variable. The best model was model 2 (*F*(10,205)=13.84, *p*<0.001), explaining 40% of the variance.

*Physical work barriers: Depression*

The models and significant predictors were identical to the models with the total score as the dependent variable. The best model was model 2 (*F*(10,205)=13.70, *p*<0.001), explaining 40% of the variance.

*External work barriers: Anxiety*

Model 2 was selected as the best model (see Supplementary Table 9), *(F*(10,205)=6.87, *p*<0.001), *R*^2^=0.25. MS-related disability and anxiety were significantly related to external work barriers.

Supplementary Table 9. Summary of multiple regression analysis with anxiety predicting external work barriers (Model 2)

| External work barriers |  |  |  |  |  |
| --- | --- | --- | --- | --- | --- |
| Variable | B | LB | UB | β | *p*-value |
| Constant | 21.53 | 15.94 | 27.13 |  | **0.001** |
| Age | -0.04 | -0.33 | 0.24 | -0.02 | 0.771 |
| MS-related disability (EDSS) | 2.82 | 0.91 | 4.73 | 0.19 | **0.004** |
| Gender male | 0.20 | -5.28 | 5.68 | 0.01 | 0.943 |
| Education middle | -0.84 | -7.39 | 5.71 | -0.02 | 0.800 |
| Education high | -2.80 | -9.53 | 3.94 | -0.08 | 0.414 |
| Disease duration | -0.04 | -0.43 | 0.34 | -0.02 | 0.822 |
| Anxiety | 1.99 | 1.21 | 2.77 | 0.37 | **0.001** |
| Avoidance-oriented coping | 0.19 | -0.07 | 0.44 | 0.09 | 0.152 |
| Emotion-oriented coping | 0.18 | -0.07 | 0.43 | 0.10 | 0.158 |
| Task-oriented coping | 0.12 | -0.15 | 0.38 | 0.06 | 0.385 |

*External work barriers: Depression*

The models and significant predictors were identical to the models with the total score as the dependent variable. The best model was model 2 (*F*(10,205)=9.09, *p*<0.001), explaining 31% of the variance.
